# Atorvastatin ameliorates early brain injury through inhibition of apoptosis and ER stress in a rat model of subarachnoid hemorrhage

**DOI:** 10.1042/BSR20171035

**Published:** 2018-06-12

**Authors:** Wentao Qi, Demao Cao, Yucheng Li, Aijun Peng, Youwei Wang, Kai Gao, Cunshan Tao, Yongkang Wu

**Affiliations:** Department of Neurosurgery,The Affiliated Hospital of Yangzhou University, Yangzhou University, Yangzhou 225001, China

**Keywords:** apoptosis, atorvastatin, caspase-3, subarachnoid hemorrhage

## Abstract

Aneurysmal subarachnoid hemorrhage (SAH) is a severe cerebrovascular disease with very poor prognosis. The aim of the present study was to evaluate the protective effects of atorvastatin on early brain injury (EBI) after SAH using a perforation SAH model. Male Sprague–Dawley rats were randomly divided into four groups: the sham group, the SAH group (model group), SAH + 10 mg.kg^−1^.day^−1^ atorvastatin (low atorvastatin group), and SAH + 20 mg.kg^−1^.day^−1^ atorvastatin (high atorvastatin group). Atorvastatin was administered orally by gastric gavage for 15 days before operation. At 24 h after SAH, we evaluated the effects of atorvastatin on brain water content, apoptosis by TUNEL assay and scanning electron microscope (SEM), and the expression of apoptosis-related proteins by immunofluorescence and Western blotting analysis. Compared with the sham group, we observed increased brain water content, significant apoptosis, and elevated levels of apoptosis-related proteins including caspase-3, CCAAT enhancer-binding protein homologous protein (CHOP), the 78-kDa glucose-regulated protein (GRP78), and aquaporin-4 (AQP4) in the SAH group. Atorvastatin administration under all doses could significantly reduce brain water content, apoptosis, and the expression levels of caspase-3, CHOP, GRP78, and AQP4 at 24 h after SAH. Our data show that early treatment with atorvastatin effectively ameliorates EBI after SAH through anti-apoptotic effects and the effects might be associated inhibition of caspase-3 and endoplasmic reticulum (ER) stress related proteins CHOP and GRP78.

## Introduction

Aneurysmal subarachnoid hemorrhage (SAH) following a ruptured intracranial aneurysm accounts for 3–7% of all strokes [1]. It affects approximately 1 per 10000 persons annually in North America, and is associated with a high morbidity and mortality [1]. A growing body of evidence suggests that early brain injury (EBI) occuring within 72 h after SAH is the primary cause of mortality in patients with SAH [2]. Apoptosis and the development of brain edema are two major pathological processes in EBI. Despite advances in diagnostic techniques and treatment, effective therapeutic interventions are still limited.

Statins, as 3-hydroxy-3-methylglutaryl coenzyme-A (HMG-CoA) reductase inhibitors, belong to a well-established class of drugs that can reduce cholesterol synthesis and increase the uptake of low-density lipoproteins. With the development for treating hypercholesterolemia, increasing evidence show that statins have a variety of pleiotropic effects, including modulating angiogenesis, regulating cell growth and apoptosis, down-regulating inflammatory mediators, reversal of endothelial dysfunction, and neuroprotective effects [3]. Studies suggest a positive impact of statin therapy on SAH. An association between statin treatment and a decrease in incidence of vasospasm after SAH has been identified in clinical studies [4,5]. A recent meta-analysis involving 2148 subjects found that statin treatment was effective in preventing delayed ischemic neurologic deficits (DINDs) in patients with SAH [6]. Futhermore, randomized controlled phase II studies demonstrated that acute initiation of statin therapy directly after SAH not only decreased the occurence of radiologic vasospasm and clinical signs of DINDs, but also reduced mortality [7,8].

Apoptosis is the most common proposed mechanism for the development of EBI. The pathways involved in apoptosis after SAH include caspase-dependent and -independent pathways, mitochondrial pathways, and cell-death receptor pathways [9]. Several studies have found that atorvastatin inhibits apoptosis and ameliorates EBI after SAH [10,11], but the underlying mechanisms remain poorly understood, especially for role of endoplasmic reticulum (ER) stress in effect of atorvastatin on SAH. The aim of the present study was to evaluate the protective effects of atorvastatin on EBI after SAH, and to assess protein expression levels of apoptosis-related proteins caspase-3 and aquaporin-4 (AQP4), and ER stress related proteins, CCAAT enhancer-binding protein homologous protein (CHOP), and the 78-kDa glucose-regulated protein (GRP78). In addition, morphological changes after atorvastatin treatment were evaluated using scanning electron microscope (SEM).

## Materials and methods

### SAH rat model and grouping

Male Sprague–Dawley rats weighing 300–350 g (bought from the Affiliated Hospital of Yangzhou University) were anesthetized with 2.0% isoflurane through a face mask. A rat perforation model of SAH was induced as previously described [28]. SAH was confirmed in each rat at autopsy. Atorvastatin was dissolved in 1% DMSO and further diluted in physiological buffer solution (PBS, final 0.01% DMSO). Atorvastatin was administered orally by gastric gavage for 15 days before operation; 10 and 20 mg.kg^−1^.day^−1^ of atorvastatin were selected because previous studies showed that atorvastatin at these doses had beneficial effects on stroke prevention [29]. The rats were randomly assigned into four groups: the sham group subjected to a similar procedure as SAH group but without perforation (*n*=18); the SAH group (*n*=18); low atorvastatin group in which SAH treated with 10 mg.kg^−1^.day^−1^ atorvastatin (*n*=18); and high atorvastatin group in which SAH treated with 20 mg.kg^−1^.day^−1^ atorvastatin (*n*=18). The animals were killed 24 h after the operation and all end points in the present study were investigated at 24 h after SAH. All experimental protocols were approved by the Ethics Committee for the Use of Experimental Animals in the Affiliated Hospital of Yangzhou University.

### Brain water content

The rats were killed at 24 h after SAH induction, and the brain water content was assessed. Briefly, the brains were quickly removed and weighed immediately (wet weight). The samples were then heated in an oven at 105°C for 24 h, and the sample brains were measured again (dry weight). The percentage of brain water content was calculated as ((wet weight – dry weight)/wet weight) × 100%. Six rats were used in each group.

### Histology and TUNEL staining assay

Rats (*n*=3 in each group) were killed 24 h after surgery, and perfused intracardially with PBS (pH 7.4) followed by perfusion with 4% paraformaldehyde (pH 7.4). Their brains were collected and placed in the same fixative at 4°C for 2 days. The brains were then frozen in tissue-freezing media and cut into 10-μm sections. TUNEL staining was performed according to the manufacturer’s protocol (Roche Inc, Basel, Switzerland) and examined under a laser scanning confocal microscope (LSM-710; Zeiss).

### Immunofluorescence assay

Double fluorescence labeling was performed as described previously [30]. Amongst the stored brains after India Ink angiography, 12 brains were randomly used from groups sham (*n*=3), SAH (*n*=3), low atorvastatin group (*n*=3), and high atorvastatin group (*n*=3), respectively. The intracranial internal carotid artery (ICA) was sectioned every 200 μm. Ten micrometers thick coronal sections were cut by a cryostat and incubated overnight at 4°C with the rabbit anti-Caspase 3 (1:50; Santa Cruz Biotechnology, Santa Cruz, CA) and rabbit anti-CHOP (1:500; Sigma-Aldrich, St. Louis, MO) antibodies, followed by incubation with appropriate fluorescence dye-conjugated secondary antibodies (Jackson ImmunoResearch, West Grove, PA). The sections were visualized with a fluorescence microscope, and pictographs were recorded and analyzed with MagnaFire SP 2.1B software (Olympus, Melville, NY).

### Western blot analysis

Protein extraction from each group was obtained by gently homogenizing in 1% PMSF and RIPA lysis buffer (50 mM Tris/HCl pH 7.4, 150 mM NaCl, 1% NP-40, 0.1% SDS). Protein lysates were mixed with SDS/PAGE loading buffer and boiled at 100°C for 5 min prior to electrophoresis. Equal amounts of protein samples were loaded on to the SDS/PAGE gel. After being electrophoresed and transferred to a PVDF membrane (Millipore, U.S.A.), the membrane was then blocked and incubated with rabbit anti-mouse polyclonal primary antibodies at a dilution of 1:1000 overnight. Primary antibodies used were acquired from Abgent: GRP78, Caspase-3, CHOP, AQP4, and GAPDH. The day after, the membranes were incubated with the corresponding secondary antibodies for 1 h at room temperature. Proteins were visualized using ECL reagent (Advansta, U.S.A.), and the bands were obtained by GeneGnome 5 (Synoptics Ltd., U.K.).

### SEM

After air drying, 1 mm × 1 mm^3^ section of tissues was sputter-coated with gold palladium particles under vacuum using Dentum Vacuum Desk II. Samples were viewed at 10.0 kV with a Hitachi Model S-4700 SEM. For cross-section inspection, grain samples from nine growth stages as described above were harvested and fixed immediately in 4% glutaraldehyde in cacodylate buffer (0.1 M, pH 7.4) at 4°C for approximately 7 days until the solution was thoroughly penetrated into the kernel. The samples were then dehydrated through an ethanol concentration series (30, 50, 70, 80, 85, 90, 95, and 100% v/v) and incubated twice in each solution for 10 min.

### Statistics

Data were expressed as mean ± S.D. Statistical differences between atorvastatin and other groups were compared by one-way ANOVA and then the Tukey–Kramer multiple comparison procedure, if a significant difference had been determined by ANOVA. A *P*-value of less than 0.05 was assumed to be statistically significant.

## Results

### Atorvastatin decreased the brain water content induced by SAH

**Figure 1:**
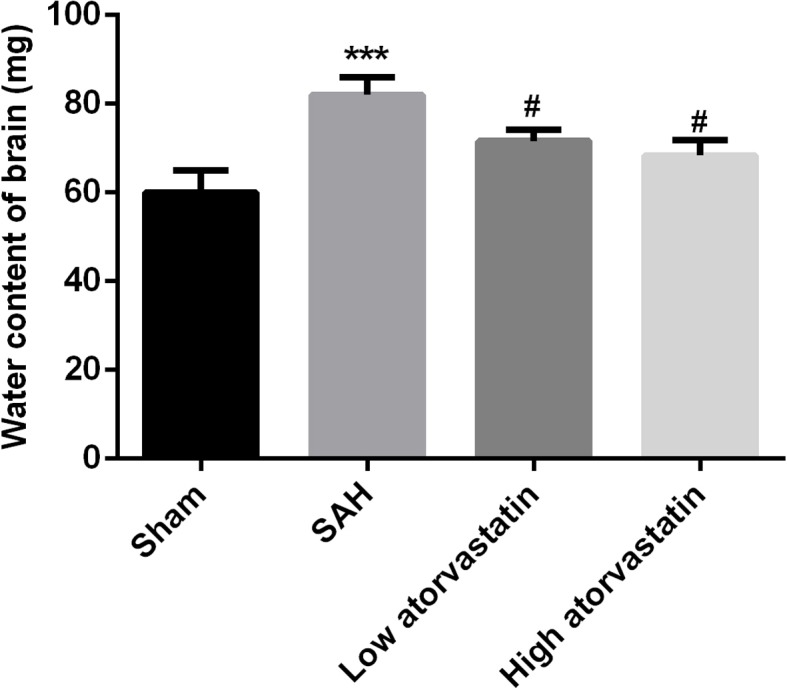
Brain water content of the four groups (six animals in each) ***P<0.001 compared with the sham group; ^#^*P*<0.05, compared with the SAH group.

At 24 h after SAH, brain water content was significantly increased in SAH group compared with that in the sham group (*P*<0.05). However when treated with atorvastatin, the amount of brain water content significantly decreased in both low and high atorvastatin groups compared with the SAH group (*P*<0.05, Figure 1) and the effect was dose dependent.

### Atorvastatin inhibited SAH-induced cell apoptosis and expression of caspase-3 and CHOP

**Figure 2:**
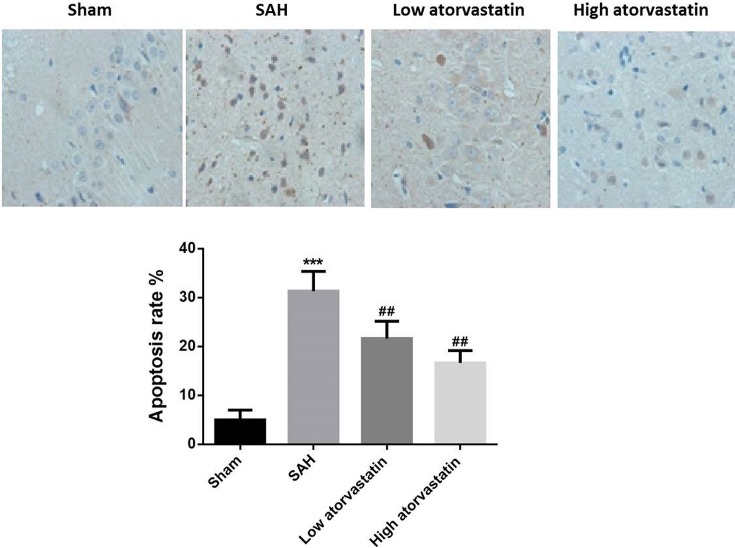
TUNEL staining of ICA after SAH Few apoptotic cells were observed in sham operated control rats (three animals in each). Quantitative analysis showed an obvious reduction in the number of apoptosis cells (number/mm^2^) by atorvastatin compared with the sham group (*P*<0.01). ***P<0.001 compared with the sham group; ^##^*P*<0.01, compared with the SAH group.

**Figure 3:**
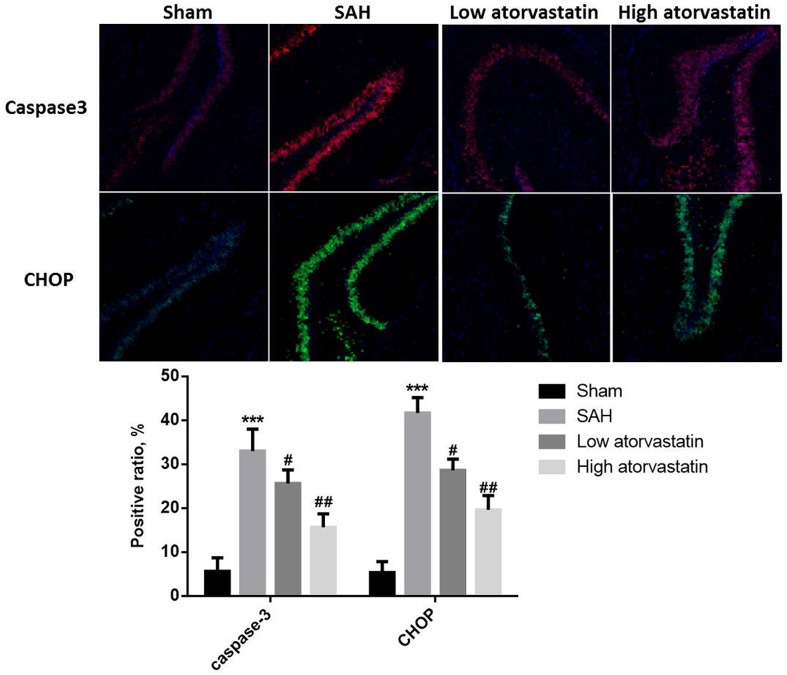
Immunofluorescence for Caspase-3 and CHOP in the intracranial ICA at 24 h after SAH (three animals in each) ***P<0.001 compared with the sham group; ^##^*P*<0.01, compared with the SAH group.

In the sham group, no TUNEL-positive cells were detected. TUNEL-positive cells were observed and increased significantly in the intracranial ICA after 24 h treated with SAH compared with the sham group (*P*<0.05, Figure 2). After treatment with atorvastatin, the TUNEL-positive cells were significantly decreased compared with the SAH group, and the effects were dose dependent (*P*<0.05). The measurement by immunofluorescence analysis showed that the levels of both caspase-3 and CHOP were significantly increased in SAH group compared with the sham group (*P*<0.05, Figure 3). And atorvastatin pretreatment in both low and high dose groups significantly reduced caspase-3 expression as well as CHOP expression in the ICA compared with the SAH group.

### Atorvastatin inhibited injury of ICA and SAH-induced increase in apoptosis-related and ER stress-related proteins

**Figure 4:**
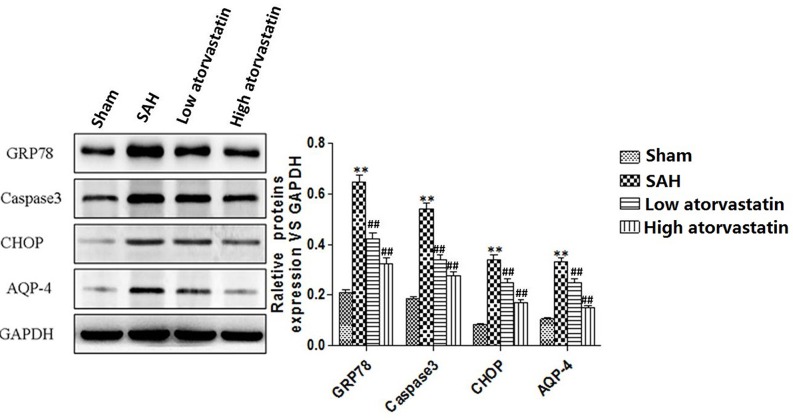
Western blot for expression of GRP78, Caspase-3, CHOP, and AQP4 Atorvastatin reduced the expression of cleaved GRP78, caspase-3, CHOP, and AQP4 (*P*<0.05 compared with the SAH group). Each column represents three independent experimental results. ***P<0.001 compared with the sham group; ^##^*P*<0.01, compared with the SAH group.

**Figure 5:**
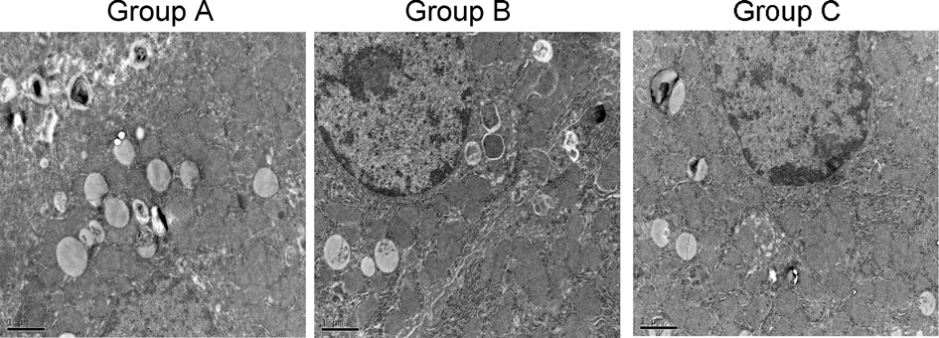
Microscopic images of intracranial ICA with SEM Scale bar was 1 μm.

Apoptosis-related proteins and ER stress related proteins were examined in ICA by Western blotting. Elevated levels of cleaved GRP78, caspase-3, CHOP, and AQP4 were observed in after SAH compared with the sham group (*P*<0.05). Both low and high doses of atorvastatin could significantly reduce the expression of cleaved GRP78, caspase-3, CHOP, and AQP4 compared with the SAH group (*P*<0.05) and the effect was dose dependent (Figure 4). SEM analysis showed broken nucleus and apoptotic body in SAH group. A series of characteristic morphological changes in cell apoptosis were observed in both low and high doses of atorvastatin groups, including cell shrinkage, smaller volume, more physalides in cytoplasm, and chromatin condensation (Figure 5).

## Discussion

The mechanism of EBI after SAH is complicated and involves the rapid rise of intracranial pressure and reduction in cerebral perfusion pressure, apoptosis, necrosis, autophagy, BBB disruption, oxidative and nitrosative stress, and inflammation [12]. The effectiveness of many agents has been studied on the inhibition of EBI after SAH, and only those that act on multiple pathways have promising effects. In the present study, we demonstrated that atorvastatin, when administered prophylacticly, ameliorated EBI after experimental SAH. Atorvastatin administration inhibited apoptosis-related proteins; the neuroprotective effects may be involved in its potential anti-apoptosis mechanisms. Caspases-3 was involved in cell apoptosis after SAH, and beneficial results were obtained when caspase activitiy was inhibited during SAH [13]. This study showed that early treatment with atorvastatin down-regulated caspase-3 expression, reduced cell apoptosis, and improved neurological outcome at 24 h after SAH, and the effects might be also associated with inhibition of ER stress. These findings support the hypothesis that atorvastatin may also work as an anti-apoptotic agent to improve neurological outcome in rats subjected to SAH.

Despite intense research efforts in SAH, we currently possess a very limited understanding of the underlying mechanisms that result in brain injury [14]. However, a number of studies have recently indicated that apoptosis may be a major player in the pathogenesis of secondary brain injury after SAH [15]. As a result, the apoptotic cascades present a number of potential therapeutic opportunities that may ameliorate secondary brain injury. Experimental data suggest that these cascades occur very early after initial insult and may be related directly to physiologic sequela commonly associated with SAH [13]. In accordance with previous reports, our data showed that the number of TUNEL-positive cells was significantly increased in SAH rats, and atorvastatin administered ahead markedly inhibited apoptosis after SAH. Apoptosis requires activation of caspases, a cascade of cysteine proteases activated by proteolytic cleavage, which target key homeostatic and structural proteins, such as actin and fodrin as well as nuclear proteins like poly (ADP-ribose) polymerase (PARP), leading to DNA fragmentation and cell death. Caspase-3 activation is considered one of the final steps responsible for apoptosis [15]. Caspase-3 can proteolytically cleave several crucial cellular proteins inducing the characteristic changes associated with apoptosis including PARP-1, which was considered a hallmark biochemical characteristic of apoptosis. Atorvastatin significantly reduced the increased caspase-3 protein expression 24 h after SAH. Although several studies have shown that statins may cause apoptosis in different cell lines including neuronal cells [16,17]. Besides, AQP-4 was also proven to be associated with cell apoptosis induced by cerebral ischemia –reperfusion injury [18]. The expression of AQP-4 was also proven to be significantly up-regulated in cerebral edema under hypobaric hypoxia and to inhibit its expression could lead to preventative effect [19]. Our data show that administration of atorvastatin to rats reduces the caspase-dependent apoptotic signal induced by SAH. Our results were in agreement with a previous study which reported that pravastatin reduced apoptosis in the hippocampus of adult rats after transient forebrain ischemia [20]. The reason for the discrepancy between *in vitro* and *in vivo* studies evaluating the effects of statins on apoptosis is unclear. One possibility is that statins do not exert a pro-apoptotic effect on brain cells *in vivo*. This hypothesis is supported by the observation that caspase-3 activation and PARP cleavage were reduced in simvastatin-treated control animals or in the contralateral side of simvastatin-treated ischemic animals [21]. Another possibility is that the pro-apoptotic effects on inflammatory cells and the inhibition of leukocyte function induced by statins overcome their pro-apoptotic effects on neuronal and/or glial cells, leading to neuroprotection.

Our study showed that atorvastatin administration significantly decreased the expression of CHOP at 24 h after SAH, which may in part contribute to its protective effects. Researches show that when ER stress becomes severe and prolonged, it can induce cell apoptosis and several proteins such as CHOP and GRP78 are involved in this process. ER stress activates CHOP and GRP78, which leads to apoptosis through several mechanisms including down-regulation of anti-apoptotic B-cell lymphoma-2 (bcl-2) protein [22]. CHOP-dependent apoptosis also requires induction of bcl-2 interacting mediator of cell death (bim) – one of the most powerful pro-apoptotic BH3-only member of bcl-2 family [23]. CHOP acts through direct transcriptional induction of bim [24,25]. Studies have shown that upon induction of ER stress, bim translocates to the ER and promotes activation of caspases [26]. In the apoptotic machinery CHOP induces bim and represses bcl-2 [27]. In the brain vessels with CHOP siRNA treatment, bim was reduced while bcl-2 rebounded, which resulted in the anti-apoptotic effect. Bcl-2 can play anti-apoptotic role at the level of mitochondria and ER where it reduces Ca^2+^ content or, alternatively, it blocks Ca^2+^ release upon cellular stress [27]. Furthermore, overexpression of bcl-2 reduces capacitative calcium entry, the important mechanism of vasospasm alone [28]. The increased bcl-2 has been shown to reduce endothelial apoptosis and cerebral vasospasm in the rabbit model of SAH treated with EPO [29].

In conclusion, our study demonstrated that early treatment with atorvastatin significantly reduced brain water content, apoptosis, and the expression levels of apoptosis-related proteins caspase-3 and AQP4, and ER stress related proteins CHOP and GRP78 at 24 h after SAH. Atorvastatin may ameliorate EBI through anti-apoptotic effects.

## Abbreviations

AQP4: aquaporin-4
BBB: blood brain barrier
bcl-2: B-cell lymphoma-2
bim: bcl-2 interacting mediator of cell death
CHOP: CCAAT enhancer-binding protein homologous protein
DIND: delayed ischemic neurologic deficit
EBI: early brain injury
EPO: erythropoietin
ER: endoplasmic reticulum
GRP78: 78-kDa glucose-regulated protein
ICA: internal carotid artery
PARP: poly (ADP-ribose) polymerase
PBS: physiological buffer solution
SAH: subarachnoid hemorrhage
SEM: scanning electron microscope
