# Expression of Concern: Atorvastatin ameliorates early brain injury through inhibition of apoptosis and ER stress in a rat model of subarachnoid hemorrhage

**DOI:** 10.1042/BSR-2017-1035_EOC

**Published:** 2024-08-22

**Keywords:** apoptosis, atorvastatin, caspase-3, subarachnoid hemorrhage

The Editorial Office has been made aware of potential issues surrounding the scientific validity of this paper “Atorvastatin ameliorates early brain injury through inhibition of apoptosis and ER stress in a rat model of subarachnoid hemorrhage” DOI: 10.1042/BSR20171035, hence has issued an expression of concern to notify readers whilst the Editorial Office investigates. It has been noted that the SAH Caspase3 and Sham CHOP panels of Figure 3 share unexpected similarities. The authors have been contacted regarding the concerns raised, but so far have not responded to the Journal.

